# Empirical Comparison of Post-processing Debiasing Methods for Machine Learning Classifiers in Healthcare

**DOI:** 10.1007/s41666-025-00196-7

**Published:** 2025-03-20

**Authors:** Vien Ngoc Dang, Víctor M. Campello, Jerónimo Hernández-González, Karim Lekadir

**Affiliations:** 1 Departament de Matemàtiques i Informàtica, Universitat de Barcelona, Barcelona, Spain; 2 Departament d’Informàtica, Matemàtica Aplicada i Estadística, Universitat de Girona, Girona, Spain; 3 Institució Catalana de Recerca i Estudis Avançats, ICREA, Barcelona, Spain

**Keywords:** Algorithmic bias, Fairness, Healthcare, Machine learning classifiers, Post-processing

## Abstract

Machine learning classifiers in healthcare tend to reproduce or exacerbate existing health disparities due to inherent biases in training data. This relevant issue has brought the attention of researchers in both healthcare and other domains, proposing techniques that deal with it in different stages of the machine learning process. Post-processing methods adjust model predictions to ensure fairness without interfering in the learning process nor requiring access to the original training data, preserving privacy and enabling the application to any trained model. This study rigorously compares state-of-the-art debiasing methods within the family of post-processing techniques across a wide range of synthetic and real-world (healthcare) datasets, by means of different performance and fairness metrics. Our experiments reveal the strengths and weaknesses of each method, examining the trade-offs between group fairness and predictive performance, as well as among different notions of group fairness. Additionally, we analyze the impact on untreated attributes to ensure overall bias mitigation. Our comprehensive evaluation provides insights into how these debiasing methods can be optimally implemented in healthcare settings to balance accuracy and fairness.

**Supplementary Information:**

The online version contains supplementary material available at 10.1007/s41666-025-00196-7.

## Introduction

Implementing machine learning (ML) in healthcare presents significant ethical challenges, as these models can inadvertently reinforce and amplify existing health disparities [1]. Chest X-ray classifiers for detecting diseases, for instance, have been found to underdiagnose conditions in underserved populations like Black females, potentially leading to treatment delays and worsening health inequalities [2]. Similarly, ML models for depression prediction show biases, exhibiting significant disparities in the true positive rate among different demographic groups, such as ethnicity and socioeconomic status [3]. Additionally, Chen et al. [4] highlighted disparities in error rates among racial groups in ML models predicting patient mortality, leading to unequal healthcare outcomes.

Bias in ML models can be described as the systematic discrepancies in model performance across different population subgroups. These discrepancies occur due to multiple factors, including representation and outcome base rate imbalances. Representation bias arises when there is a lack or overrepresentation of certain groups within the training data. This imbalance causes the model to learn either too little or too much about these groups, leading to skewed predictions. For example, Park et al. [5] found that ML models predicting postpartum depression significantly underdiagnosed Black women due to their underrepresentation in the training data, leading to inadequate medical care. Disparities in base rates of outcomes, which refer to differences in the prevalence of specific outcomes across different groups, also contribute to bias. These disparities can lead the model to learn that one group has a higher probability of being classified into a certain class than another. For instance, prior research shows that the prevalence of depression differs across sex subgroups; women are about twice as likely as men to develop depression during their lifetime [6]. In a study on depression prediction [3], this prevalence difference led to models with a significantly higher true positive rate for females than for males. With such a model, females are more likely to be correctly identified as needing treatment, while males may be underdiagnosed, leading to unequal access to necessary care.

To address these issues, many debias techniques have been developed, which attempt to improve various fairness criteria by acting on different stages of the ML process [7]. So-called pre-processing techniques adjust the values of the training data to remove disparities between population groups, and in-processing techniques modify the learning algorithms (note that this is model specific) to avoid them being impacted by bias. However, pre-processing methods require access to raw training data, which may not always be feasible due to privacy concerns, particularly in healthcare applications. In-processing methods, on the other hand, integrate fairness constraints directly into the model training process. While effective in many cases, these methods require modifying the learning algorithm itself, making them unusable with pre-trained models and often requiring retraining. This limits their applicability in real-world clinical settings, where retraining can be costly, time-consuming, and technically challenging. The third family of debias techniques, the post-processing methods, adjust the model’s predictions to ensure fairness. This approach does not require access to the training data, which preserves privacy. Moreover, these methods do not modify the learning process and work with any type of already-trained models. This simplifies the use of mitigation techniques in existing systems without the need for model retraining. The present study focuses on this latter class of debiasing methods.

In this study, the research question we address is how effective current post-processing debiasing methods are at mitigating bias in unfair ML classifiers used in the clinical setting. Specifically, we aim to study how different data characteristics affect debiasing performance, how different debiasing methods balance fairness and predictive accuracy, as well as different fairness notions, and how they affect untreated protected attributes’ fairness. We also strive to characterize how these methods perform across model types and datasets. To answer this question, we empirically study a range of state-of-the-art post-processing debiasing methods across a diverse set of real-world datasets, from healthcare, but also other domains, to assess the broader applicability of these methods across different types of data distributions and biases. Synthetic data with varying levels of class separation and unprivileged representation is used to test these methods in a wide range of biased scenarios. By examining the frontier of achievable accuracy and fairness levels, we aim to provide insights into the strengths and weaknesses of each method, focusing on the trade-offs between group fairness, which ensures that the model performs similarly across different subgroups according to a certain statistical metric, and predictive performance (type-1). Additionally, we explore trade-offs among different notions of group fairness (type-2) and their impact on untreated attributes. While previous studies have explored post-processing debiasing methods in healthcare settings [3, 5], this comprehensive evaluation goes further by seeking to elucidate the practical implications of using each post-processing debiasing method, providing a detailed understanding of how these techniques can be optimally applied to balance accuracy and fairness across different healthcare datasets. To the best of our knowledge, our study is among the first to compare multiple post-processing debiasing methods across diverse healthcare scenarios empirically. The main contributions are:

- A systematic evaluation of seven post-processing debiasing methods using both real-world healthcare and synthetic data, covering a wide range of unfair scenarios, ML models, and competing fairness objectives.
- The identification of relevant dataset characteristics, such as class distribution and unprivileged group representation, and their influence on the effectiveness of some post-processing debiasing methods.
- The characterization of unintended increases in disparities regarding untreated protected attributes when targeting a different one depending on the method and influenced by the negative correlation between attributes.
- A list of practical recommendations for clinical-domain practitioners to select the appropriate post-processing debiasing method based on dataset composition and fairness objectives.

## Related Work

A plethora of post-processing debiasing methods have been proposed to mitigate bias in machine learning models, but most of them have not been extensively tested on clinical datasets, raising concerns about their applicability in real-world medical decision-making [8, 9]. Chen et al. [10] conducted a key empirical study of 17 debiasing methods across eight datasets, but included only one medical dataset and tested a limited number of post-processing methods, restricting its applicability to clinical settings. Similarly, other empirical studies such as the one by Biswas and Rajan [11] have primarily focused on general-purpose datasets, leaving healthcare-specific fairness concerns unexplored.

Furthermore, most studies focus on fairness regarding a single attribute (e.g., race or gender), not paying attention to possible interactions between different protected attributes [12, 13]. Recently, Chen et al. [14] assessed 11 state-of-the-art fairness improvement methods for multiple protected attributes across five datasets from financial, social, and medical domains, providing insights into multi-attribute fairness. However, the study does not examine how debiasing strategies interact with varying dataset characteristics, an essential factor to generalize the results to different healthcare applications.

To this respect, existing empirical fairness studies in healthcare tend to focus on specific medical domains, rather than systematically comparing debiasing strategies across diverse clinical tasks. For example, Zhang et al. [15] analyzed fairness in chest X-ray classification, benchmarking nine debiasing methods across different fairness definitions for detecting pneumothorax and fractures. Similarly, Marcinkevics et al. [16] studied post-processing fairness techniques within chest X-ray analysis, focusing on pneumonia and enlarged cardiomediastinum detection. Soltan and Washington [17] assessed post-processing methods for breast cancer stage classification, while Dang et al. [3] conducted an empirical comparison of debiasing techniques for depression prediction across multiple study populations. Additionally, Park et al. [5] evaluated approaches for reducing bias in ML models predicting postpartum depression. While these studies provide valuable insights into the specific domains, their conclusions barely generalize to broader healthcare applications.

Unlike prior studies, our study performs a systematic empirical comparison of post-processing debiasing methods through different healthcare datasets. By analyzing how different post-processing debiasing methods perform in varied clinical settings and data characteristics, we seek to contribute to a better understanding of their applicability and limitations in healthcare AI.

## Materials and Methods

### Datasets

Let us define a dataset for fairness analysis as $D=(A,X,Y)$, which is assumed to be an iid sample from an unknown distribution *p*(*A*, *X*, *Y*), where *A* is the protected attribute, *X* is the descriptive variable, and *Y* is the class variable. In this study, we will assume that both the protected and the class variables are binary, that is, they can only take two possible values, $\Omega _A = \{0,1\}$ and $\Omega _Y = \{0,1\}$.

This study considers eight real-world datasets: four from the medical domain and four from other domains. For the medical datasets, we use **CDC Diabetes Health Indicators** [18], **Heart Disease** [19], and **Medical Expenditure Panel Survey** (MEPS) [20]. Additionally, we accessed data from the UK Biobank [21] under the project titled “Association between Early-Life-Stress and Psycho-Cardio-Metabolic Multi-Morbidity: The EarlyCause H2020 Project” (application number 65769) for the **Depression Disease** dataset. The UK Biobank obtained ethical approval from the North West Multi-centre Research Ethics Committee (MREC) and the Community Health Index Advisory Group (CHIAG), and we adhered to the UK Biobank’s ethical guidelines by signing a Material Transfer Agreement (MTA). All participants in this study provided written informed consent. These medical datasets are crucial for evaluating fairness in healthcare applications, providing valuable insights into the impacts of biases on clinical outcomes and health equity. For the non-medical datasets, we use **Adult** [22], **German Credit** [23], **COMPAS** [24], and **Bank Marketing** [25]. These non-medical datasets are widely recognized and extensively used benchmarks in fairness research. They are frequently employed to assess and compare the performance of debiasing methods due to their diverse demographic variables and well-documented biases. A complete summary of these datasets, including sample sizes, feature counts, and distributions of protected attributes, is available in Supplementary Tables 1 and 2.

We also use **synthetic** data, generated with a procedure similar to that of [26]. This data consists of a binary protected variable $A$, a two-dimensional descriptive variable $X = (X_0, X_1)$, and a binary class variable $Y$. The descriptive feature $X$ is generated from the following 2-dimensional Gaussian distributions:

1 $$\begin{aligned} X \mid Y = 1&\sim \mathcal {N} \left( \begin{pmatrix} 2 \\ 2+s \end{pmatrix}, \begin{pmatrix} 5 &  1 \\ 1 &  5 \end{pmatrix} \right) , \end{aligned}$$

2 $$\begin{aligned} X \mid Y = 0&\sim \mathcal {N} \left( \begin{pmatrix} -2 \\ -(2+s) \end{pmatrix}, \begin{pmatrix} 10 &  1 \\ 1 &  3 \end{pmatrix} \right) \end{aligned}$$

The value of the protected attribute *A* is defined as $a = \text {sgn}(x_0)$, making it dependent on that of feature $X_0$. The parameter *s*, which determines the mean value of the descriptive feature $X_1$, allows us to vary the separability of the classes, and thus, the difficulty of the learning task.

### Evaluation Metrics

In this study, we train models from different ML families using the datasets presented above. These include logistic regression (LR) [27], a linear classifier, XGBoost (XGB) [28], a tree-based ensemble method, and deep neural networks (DNN) [29]. We evaluate their predictive and fairness performance before and after bias mitigation. These metrics allow us to assess the trade-offs between fairness and predictive performance.

#### Predictive Performance Metrics

We evaluate predictive performance using three metrics: Accuracy (Acc), Balanced Accuracy (BAcc), and F1-score. These metrics capture different aspects of model performance. BAcc accounts for sensitivity and specificity, making it suitable for imbalanced datasets. While Acc measures overall correctness, F1-score balances precision and recall to provide insight into both false positives and false negatives.

#### Fairness Metrics

We evaluate fairness performance using Equal Opportunity Difference (EOp) and Equalized Odds Difference (EOd) as proposed by [30]. EOp focuses on equality in true positive rates (TPR), ensuring that individuals at risk (e.g., patients needing medical intervention) are identified equally well across protected and unprotected groups. This is particularly crucial in medical settings where underdiagnosis in any group can lead to inadequate treatment and exacerbation of health conditions. EOp is defined as:

$$ \text {EOp} = |\text {TPR}_0 - \text {TPR}_1| $$

EOd provides a more comprehensive view of fairness by assessing the average disparity in both true-positive and false-positive rates across groups. In a medical context, this ensures not only that high-risk patients are identified correctly but also that the likelihood of false alarms (leading to further testing) is balanced across groups. EOd is defined as:

$$ \text { EOd} = \frac{1}{2} \left( |\text {FPR}_0 - \text {FPR}_1| + |\text {TPR}_0 - \text {TPR}_1| \right) $$

#### Fairness-Performance Trade-off Characterization

**Fig. 1:**
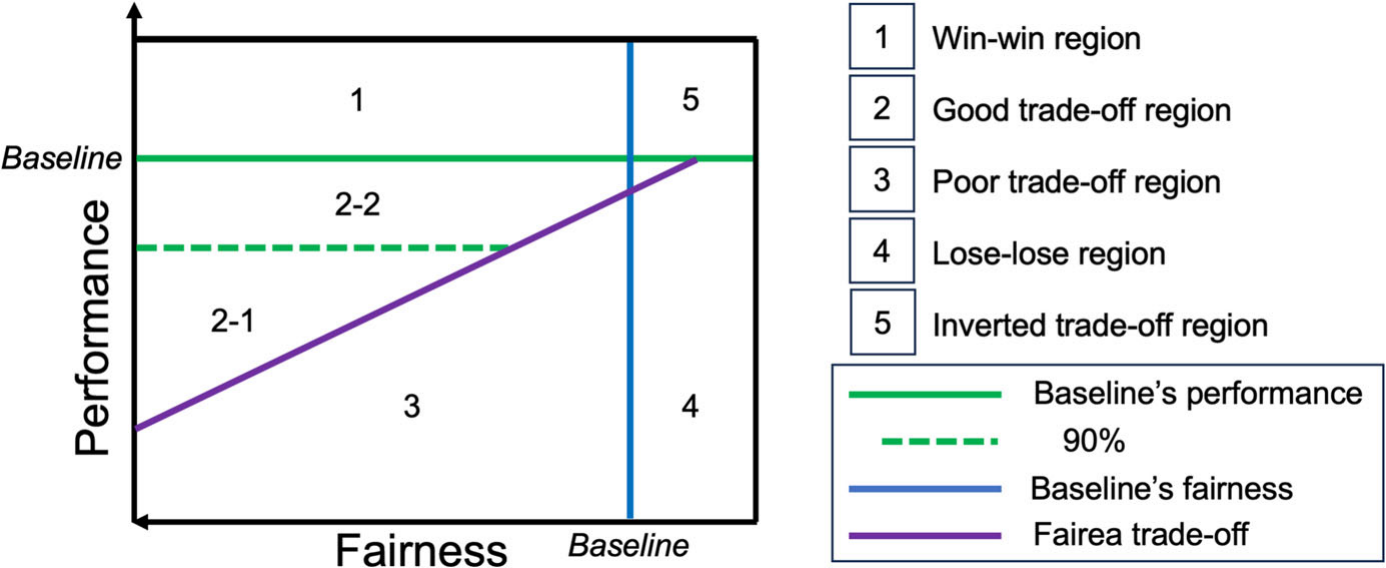
Representation of a performance vs fairness plot with a graphical description of mitigation regions of the Fairea framework. Here, the performance metric is assumed to be the larger the better, whereas the fairness metric is assumed to be the smaller the better. The diagonal purple line represents the baseline trade-off that allows for identifying acceptable mitigation outcomes

We use Fairea framework [31] to qualitatively measure and visualize fairness-performance trade-offs. A baseline trade-off is drawn by progressively modifying model predictions to simulate a range of fairness outcomes at different levels of accuracy. This baseline serves as a reference to evaluate and categorize bias mitigation results into five distinct conditions (see Fig. 1):

- **Win-win** region: Both fairness and accuracy improve.
- **Good trade-off** region: Fairness improves, and accuracy reduction remains within an acceptable range as defined by the Fairea baseline trade-off.
- **Poor trade-off** region: Fairness improves, but accuracy reduction exceeds the acceptable range.
- **Inverted trade-off** region: Fairness worsens while accuracy improves.
- **Lose-lose** region: Both fairness and accuracy worsen.

This baseline acts as the minimum standard for an acceptable trade-off. A debiasing method is considered effective only if it provides outcomes over this baseline (in the *win-win* or *good trade-off* regions). We include an additional line in Fig. 1 at 90% of performance to delimit a subarea (2.2) of the *good trade-off* region that reflects practical concerns in medical applications. With this, we indicate a preference for performance over fairness. Fairness adjustments become less acceptable if they severely reduce the ability to detect critical health outcomes. Note that this factor can vary depending on the specific application. For high-stakes contexts, a stricter factor approaching 99% may be necessary to minimize accuracy loss.

### Algorithms

We assess seven state-of-the-art post-processing debiasing methods in our study:

- **CPP** (Calibrated Equalized Odds Post-Processing) [32] calibrates predicted probabilities to equalize false-positive or false-negative rates between groups, as both cannot be equalized concurrently. In our study, recall is prioritized to minimize missed at-risk cases, leading us to equalize false-negative rates for fair and equitable predictions.
- **EPP** (Equalized Odds Post-Processing) [30] aims to optimize equalized odds by finding the optimal probability thresholds for different demographic groups.
- **ROC** (Reject Option Classification) [33] finds an optimal interval around the decision boundary to flip predicted labels to enhance fairness. In our study, this search is guided by EOp fairness metric.
- **PSTA** (Population Sensitivity-Guided Threshold Adjustment) [3] addresses bias by adapting decision thresholds for different demographic groups based on their prevalence rates in the training data. This technique improves fairness by ensuring that the sensitivity for unprivileged groups aligns with the overall population, optimizing the EOp while maintaining acceptable false-positive rates.
- **FACT** (Fairness-Confusion Tensor) [34] optimizes group-specific confusion matrices to balance accuracy and fairness. It flips prediction labels based on fairness constraints and predictive performance measurements. Acc and EOp metrics are enforced, in line with this study’s settings.
- **GSTAR** (Group Specific Threshold Adaptation for Fair Classification) [35] optimizes classification thresholds for each demographic group to balance fairness and accuracy. While addressing EOd, the default option in the available implementation, it learns thresholds based on group-specific probability distributions and confusion matrices.
- **MAB** (Multiaccuracy Boost) [36] iteratively refines a model’s predictions by identifying and correcting biases through residual errors, ensuring improved accuracy and fairness across subpopulations. MAB enhances the model’s performance without requiring explicit knowledge of the protected attribute.

The selection of these methods was driven by their diverse fairness adjustment strategies and their potential to improve fairness in healthcare applications. They cover a range of post-processing approaches, including probability calibration (CPP, MAB), group-specific threshold adaptation (GSTAR, PSTA), decision-boundary adjustments (ROC), confusion matrix optimization (FACT), and group-wise probability thresholding (EPP).

Some of these methods have already demonstrated their relevance in healthcare. CPP, EPP, and ROC are implemented in popular programming toolkits like IBM AIF360 [37], facilitating their widespread adoption and making them strong baselines for fairness interventions. PSTA and MAB have also been tested in medical domains [3, 36], demonstrating their practical utility in specific clinical applications. MAB was also chosen due to its interesting ability to improve fairness without requiring the protected attribute, making it particularly valuable when demographic data is unavailable or ethically sensitive. GSTAR and FACT have not been tested in medical applications, but their focus on threshold adaptation and confusion matrix optimization could be particularly useful in healthcare domains where decision thresholds impact patient outcomes.

With this selection, we provide a comprehensive evaluation of both well-established and emerging competitive post-processing techniques to assess their potential for improving fairness in ML models for healthcare.

### Experimental Design

We have designed two experiments to compare the debiasing methods in different scenarios. Using both real and synthetic data, we simulate different key characteristics of the data to cover a larger set of scenarios, allowing us to evaluate the effectiveness of the methods across a wide range of conditions. To ensure robust and consistent evaluation across these scenarios, we apply 5-fold cross-validation for performance assessment. The detailed configuration of ML models, including hyperparameter selection, is provided in Supplementary Tables 3 and 4. The models were trained on a GPU workstation with an 8-core Intel^®^ Xeon^®^ CPU, an NVIDIA Tesla T4 GPU, and 64 GB memory. We used Scikit-learn [38], XGBoost [28], and Keras [39] to train the models, while AIF360 [37] implementation of CPP, EPP, and ROC was employed.

#### Synthetic Data

In the first experiment, we use synthetic data to explore the performance of the debiasing methods under controlled conditions:

##### Class Separation

**Fig. 2:**
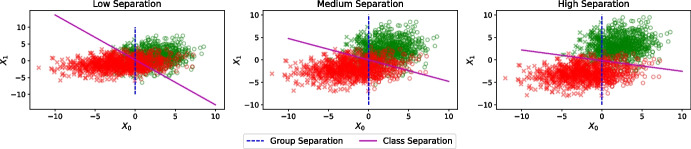
Synthetic data with low (left), medium (center), and high (right) class separation. Different markers (crosses and circles) represent different protected attribute’s values (groups). A dashed line points out the frontier between groups. Different colors (green and red) represent different classes (positive and negative, resp.). A solid line represents the best linear model that separates the classes

We evaluate the effectiveness of the seven post-processing debiasing methods in experimental scenarios with varying levels of class separation. Thus, we generate synthetic data with low, medium, and high-class separation. *Medium separation* data is generated with $s = 0$, using the parameters described in Section 3.1. To create *low separation* data, we set $s = -1$ so that the mean value of the $X_1$ feature for the positive class ($Y = 1$) is decreased by 1, and the mean value of the $X_1$ for the negative class ($Y = 0$) is increased by 1. This effectively brings the classes closer together, resulting in low separation. Conversely, to create *high separation* data, we use $s = 1$ with the opposite result: the mean value of the $X_1$ feature for the positive class is increased by 1, and the mean value of the $X_1$ feature for the negative class is decreased by 1. This effectively pushes the classes farther apart, resulting in high separation. Figure 2 illustrates the data distributions under these different separation conditions. The degree of class separation indicates the difficulty of the classification task; greater overlap between classes requires more complex models to achieve accurate classification. By evaluating on these scenarios, we assess how the complexity of the classification task affects the ability to mitigate bias for each method.

##### Attribute Imbalance

We evaluate the robustness of the bias mitigation methods through different unprivileged representation scenarios. Thus, we generate synthetic data as described in Sect. 3.1 with increasing rates of representation (10%, 30%, 50%, 70%, and 90%) of the unprivileged group. These scenarios can involve either a lack of representation or an overrepresentation of the unprivileged group. The fraction of positive cases (favorable label) is held constant at 50%. The following procedure maintains class balance while allowing us to test different rates of unprivileged group representation. We first generate a class-balanced dataset containing 2000 samples, with 1000 positive $(Y = 1)$ and 1000 negative $(Y = 0)$ instances, using a fixed random seed for reproducibility. Given the distributions in (1) and (2) with $s = 0$, the probability that $A = 0$ (i.e., $X_0 < 0$) is 0.181 for $Y = 1$ and 0.733 for $Y = 0$. To further investigate the impact of varying unprivileged group representation, we perform a 5-fold cross-validation. In each fold, the test set remains consistent across all experiments, while the training sets are adjusted to achieve different ratios of unprivileged group representation. We implemented a stratified sampling approach to (sub)sample the unprivileged and privileged groups to achieve the target representation ratio while ensuring the dataset remains class-balanced. The procedure, detailed in Algorithm 1, iteratively adjusts the sample sizes of the privileged and unprivileged groups to ensure feasibility and class balance for each target ratio. We ensure that the resulting training sets are nested, meaning that datasets with lower ratios of unprivileged representation (e.g., 10%) are subsets of those with higher ratios (e.g., 30%). This design enables us to simulate scenarios with both insufficient and excessive representation of the unprivileged group, thereby assessing the impact of these imbalances on the effectiveness of the debiasing methods.

**Algorithm 1:**
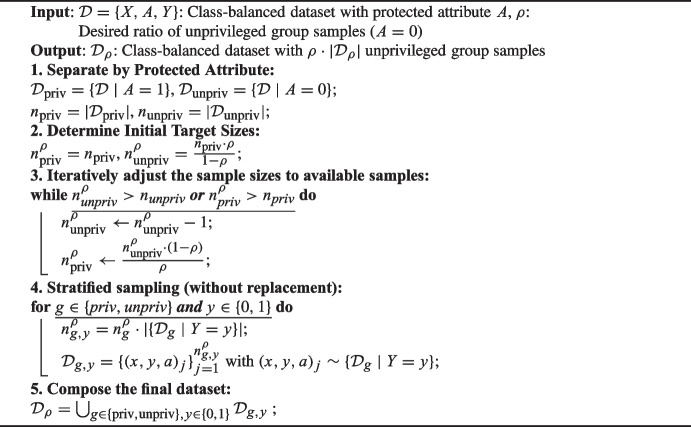
Creating a dataset with a given unprivileged group rate.

#### Real Data

In the second experiment, we use real-world data to assess the performance of the debiasing methods under naturally occurring bias conditions across various medical and non-medical datasets, focusing on varying levels of class imbalance and unprivileged group representation.

##### Class and Attribute Imbalance

**Fig. 3:**
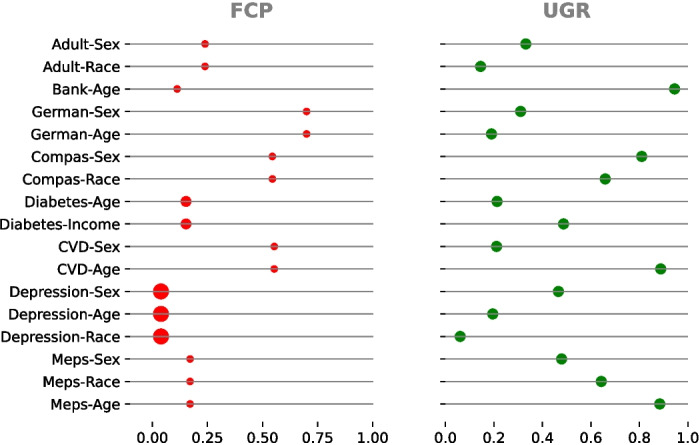
Measurement of favorable class proportion (FCP) and unprivileged group rate (UGR) across datasets. In the FCP column, the size of each dot reflects the proportionate sample size of the dataset, with larger dots indicating datasets with a greater number of samples

Each of the 8 real-world datasets has a different number of protected attributes. All the combinations of dataset and protected attribute amount to 17 different real-world case studies for bias mitigation. The favorable class proportion (FCP) and unprivileged group rate (UGR) across these scenarios are displayed in Fig. 3. We categorize these measurements into three levels: low, medium, and high. Specifically, low, medium, and high class-imbalances correspond to the intervals [0, 0.25), [0.25, 0.75), and [0.75, 1], respectively. For unprivileged group representation, we use the intervals [0, 0.20), [0.20, 0.50), and [0.50, 1], respectively.

We group the case studies according to these two characteristics. In total, we evaluate 7 distinct combinations of class and attribute imbalance, as summarized in Table 1. Medical datasets exhibit more extreme levels of class imbalance with varying levels of unprivileged group representation. In contrast, non-medical datasets typically show medium levels of both class and attribute imbalances. This variation underscores the need to assess debiasing methods under different conditions, as imbalances tend to be more varied and severe in medical datasets. Grouping case studies allows us to establish relationships with the results obtained from synthetic data, providing a comprehensive assessment of each method’s impact.

Our analysis focuses on two primary trade-offs: (type-1) between group fairness and predictive performance, and (type-2) between different notions of group fairness. In each analysis, we assess the absolute and relative changes in two performance metrics (BAcc and F1-score) and two fairness metrics (EOp and EOd) regarding treated and untreated protected attributes before and after employing debiasing methods.

**Table 1:** Summary of class and attribute imbalance levels and corresponding dataset-attribute pairs

Class imbalance degree	Unprivileged representation degree	Dataset-attribute pairs
High	High	(Bank, Age), (Meps, Race), (Meps, Age)
High	Low	(Depression, Age), (Depression, Race)
High	Medium	(Diabetes, Age), (Diabetes, Income), (Depression, Sex), (Meps, Sex)
Low	High	(Compas, Sex), (Compas, Race), (CVD, Age)
Low	Medium	(CVD, Sex)
Medium	Low	(Adult, Race), (German, Age)
Medium	Medium	(Adult, Sex), (German, Sex)

##### Impact on Untreated Protected Attributes

Additionally, we analyze the impact of bias mitigation methods on untreated protected attributes. For this analysis, we consider each combination of *(dataset, treated protected attribute, and untreated protected attribute)* as a case. We calculate the proportions of cases across different combinations of class imbalance and unprivileged group representation where applying a debiasing method to one protected attribute inadvertently affects the fairness of other unprotected attributes, then average these proportions to summarize the overall impact. To measure the effect size, we use Cliff’s $\delta$, defining a change with an absolute value of $\delta$ of 0.428 or higher as indicative of a large effect [40].

**Fig. 4:**
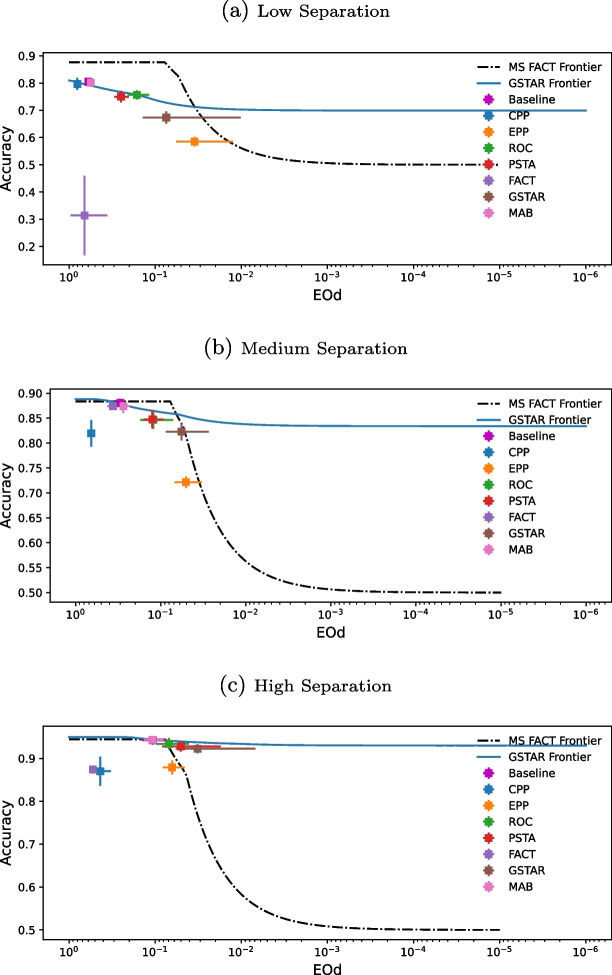
Pareto frontiers of accuracy and fairness trade-offs for post-processing debiasing methods under different class separation levels on synthetic data, using LR as the classifier. Points represent mean Acc-EOd and error bars indicate the standard deviation over 5-fold cross-validation for the baseline and the different debiasing techniques

## Results

### Influence of Data Characteristics

Here we study how different data characteristics impact the performance of post-processing debiasing methods. We generate synthetic data to simulate varying levels of class separation and unprivileged group representation. This allows us to analyze how these characteristics influence fairness-performance trade-offs. For the sake of simplicity, we present results for LR in this section. Additional results for XGB and DNN are provided in the supplementary material. The effectiveness of post-processing debiasing methods varies across models, with XGB showing weaker fairness improvements since it is inherently fairer compared to LR and DNN before bias mitigation. Despite these differences, post-processing debiasing methods exhibit consistent trends across models in their response to class separation and unprivileged representation. These findings further demonstrate the generalizability of post-processing debiasing methods across different ML models.

**Fig. 5:**
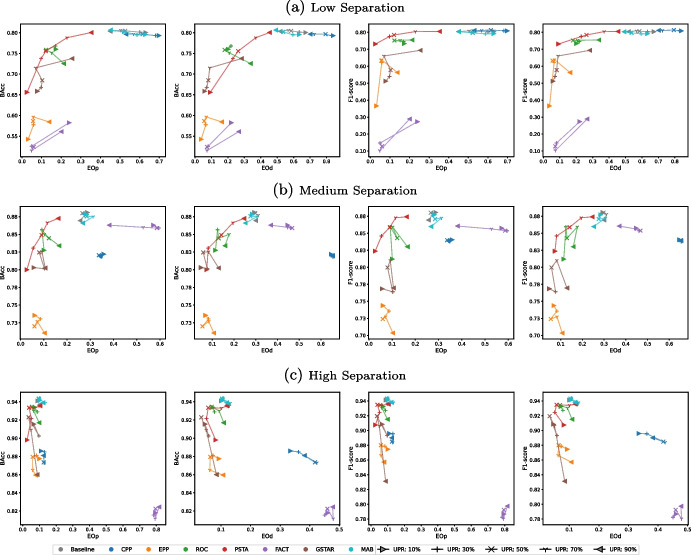
Type-1 trade-offs between performance metrics (BAcc and F1-score) and fairness metrics (EOp and EOd) achieved by a baseline LR model and, on top of it, the post-processing debiasing methods across synthetic datasets with varying levels of unprivileged representation (10%, 30%, 50%, 70%, 90%; lines connect the results of similar experiments with increasingly higher UGR) and class separation (low, medium, high; plots in different rows)

Figure 4 illustrates the fairness and performance achieved by the different debiasing techniques across synthetic datasets with varying levels of class separation: low, medium, and high. The upper right region indicates higher performance measurements (Acc) and lower bias measurements (EOd), optimizing both performance and fairness. In clinical settings, approaching this area, that is, maintaining high performance (preserving or slightly reducing Acc) while improving fairness (reducing EOd), is crucial to ensure that patient care remains effective and equitable. Figure 4 also shows the FACT [34] and the GSTAR [35] Pareto frontiers. Pareto frontiers represent the optimal trade-offs between the two competing objectives: performance and fairness. GSTAR exhibits a superior Pareto frontier across all three scenarios for post-processing debiasing methods, reflecting better trade-offs between performance and fairness. For *low separation* scenarios, PSTA and ROC are the top-performing methods, achieving a good balance between accuracy and fairness, with EPP also performing well but having a higher negative impact on performance. For *medium-class separation*, PSTA and GSTAR are the most effective, offering substantial reductions in fairness disparities while moderately impacting performance. In high *separation scenarios*, GSTAR demonstrates the best trade-off, providing superior fairness improvements with moderate performance impacts. PSTA continues to perform well, effectively reducing fairness disparities with minimal performance degradation, while ROC maintains a strong balance between accuracy and fairness across all levels of class separation. Overall, if the goal is to keep performance relatively unchanged or only slightly reduced while maximizing fairness, PSTA, ROC, and GSTAR are the recommended methods. Notably, the baseline model’s performance across different separation levels highlights the importance of employing debiasing methods, as increased class overlap significantly reduces fairness, making debiasing essential for improving equitable outcomes.

Figure 5 illustrates the fairness and performance achieved by the different debiasing techniques, using various fairness and performance metrics, across synthetic datasets with different levels of unprivileged representation and class separation. For all levels of class separation, PSTA and ROC consistently perform best at lower unprivileged representation levels, effectively reducing EOp and EOd while maintaining BAcc. GSTAR seems competitive but it shows a higher negative impact on BAcc regarding PSTA and ROC. Notably, PSTA and ROC are particularly strong in reducing EOp, whereas GSTAR is most effective in reducing EOd. FACT, CPP, and MAB tend to increase unfair disparities, negatively impacting performance. EPP reduces disparities but at a high performance cost.

As unprivileged representation increases, PSTA and ROC remain effective, while FACT, CPP, and MAB continue to underperform. MAB and CPP consistently exhibit poor performance, increasing disparities, and degrading balanced accuracy. FACT also increases disparities in medium and high separation; it only reduces disparities in low separation while compromising balanced accuracy. EPP reduces unfair disparities but generally compromises performance. Results in terms of F1-score, as shown in Fig. 5, generally follow similar trends to those observed with BAcc, especially in high and medium separation scenarios. In low separation scenarios, FACT and GSTAR experience a greater drop in F1-score, while PSTA and ROC present more stable performance. Within these results, PSTA, ROC, and GSTAR stand out in balancing fairness and accuracy across different conditions.

**Table 2:** Mean absolute and relative (in parentheses) changes — before and after debiasing using the different debiasing methods — in performance and fairness metrics averaged across ML models, and class and attribute imbalance combinations in real-world datasets

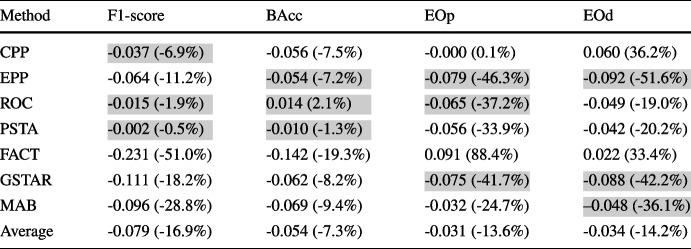	

The top three values for each metric are highlighted

### Type-1 Trade-off: Performance vs. Fairness

In this section, we explore the trade-off between predictive performance and fairness when applying post-processing debiasing methods in real-world problems. Table 2 presents for each debiasing method absolute and relative changes in performance and fairness metrics averaged across different ML models, real-world datasets, and combinations of class imbalance and unprivileged group representation. Absolute changes are calculated as the measurements after debiasing minus the baseline metric values. Thus, negative values for performance metrics indicate a decline in model performance, while negative values for fairness metrics reflect improvements. It is important to note that the phenomenon where a comparatively larger absolute change corresponds to a smaller relative change (observed for example for methods ROC-PSTA and the EOd metric), and vice versa, can be attributed to the separate averaging of absolute and relative changes across different class-attribute combinations. When these changes are averaged separately, the varying baseline values across datasets influence the relative changes, leading to disproportionate relative impacts that do not necessarily align with the absolute changes. After applying post-processing methods, F1-score consistently decreases with all the debiasing methods, and BAcc generally declines too, with a negligible increase observed when using method ROC. As expected, all methods generally decrease fairness metrics, EOp and EOd, except for FACT and CPP. Specifically, CPP shows a moderate mean negative impact on performance, with short F1-score and BAcc reductions, but it is rather inefficient in reducing unfair disparities. EPP and GSTAR also negatively impact performance but are remarkably effective in lowering unfair disparities, achieving notable reductions in EOp and EOd. ROC achieves a good balance with minimal impact on performance metrics while considerably improving fairness measurements. PSTA is competitive regarding ROC, with a different trade-off between fairness and performance (better performance results, worse fairness measurements). In contrast, FACT shows undesirable results in fairness metrics, with increases in both EOp and EOd, while also having a pronounced negative impact on performance metrics, making it less effective overall. Lastly, MAB significantly negatively impacts performance metrics but improves unfair disparities. Overall, ROC and PSTA are the most balanced approaches, effectively improving fairness with minimal impact on performance metrics.

Table 3 presents for each debiasing method the absolute and relative changes in performance and fairness metrics, averaged across ML models and real-world datasets with rough class balance separated by unprivileged representation degree. It mirrors the class-balanced settings of the synthetic datasets in Fig. 5, allowing us to examine the consistency of findings obtained from synthetic data with those from real-world datasets. With synthetic data, Fig. 5 shows that methods like PSTA and ROC effectively reduce fairness disparities with minimal impact on performance metrics across different levels of unprivileged representation. Similarly, Table 3 demonstrates that PSTA and ROC maintain this balance in real-world datasets, reducing EOp and EOd significantly while having minimal negative impact on BAcc and F1-score. GSTAR and EPP are also competitive in both synthetic and real-world settings but show a higher negative impact on performance (F1-score). Conversely, methods like FACT and CPP increase unfair disparities and degrade performance in both types of datasets. MAB does not significantly impact performance but it is not consistent in reducing unfair disparities. The consistency of the results from synthetic data with those from real-world datasets provides strong evidence of the robustness of PSTA and ROC in mitigating bias across different levels of unprivileged representation. Detailed results, including those with high and medium class imbalance for real-world scenarios, broken down into non-medical and medical categories, are available at https://github.com/ngoc-vien-dang/FairML4H-PostProcessing.

**Table 3:** Mean absolute and relative (in parentheses) changes –before and after debiasing using the different debiasing methods– in performance and fairness metrics averaged across ML models in real-world datasets with low-class imbalance

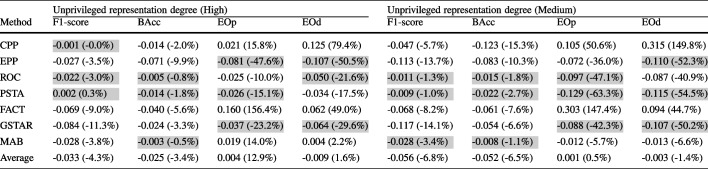	

Each subtable shows results separated by unprivileged representation degree. The top three values for each metric are highlighted

**Table 4:** Mean absolute and relative (in parentheses) changes — before and after debiasing using the different debiasing methods — in performance and fairness metrics averaged across ML models in real-world datasets separated for non-medical and medical datasets

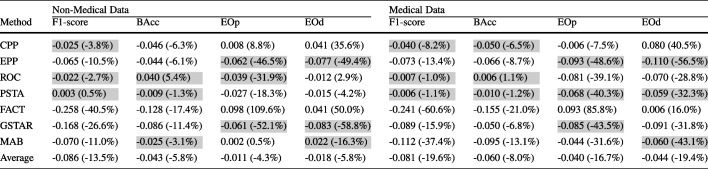	

The top three values for each metric are highlighted

**Table 5:** Mean absolute and relative (in parentheses) changes — before and after debiasing using the different debiasing methods — in performance and fairness metrics averaged across ML models for medical datasets with high-class imbalance separated by unprivileged representation degree

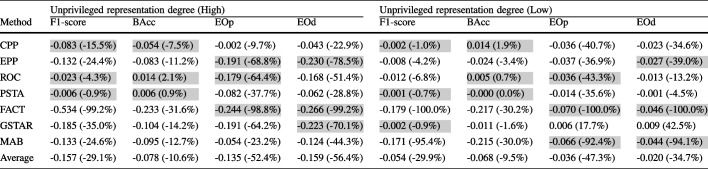	

The top three values for each metric are highlighted

As described in Table 4, ROC and PSTA effectively balance performance and fairness in medical contexts. In non-medical contexts, PSTA maintains consistent performance and fairness improvements, while ROC preserves performance, achieving strong EOp reduction with a slight relative increase in EOd. Specifically, MAB shows limited fairness improvement in non-medical scenarios and suffers severe performance degradation in medical datasets. Meanwhile, GSTAR performs better in medical contexts. Overall, methods tend to have a less negative impact on performance in non-medical contexts compared to medical contexts.

Classification tasks on balanced datasets are generally considered easier than on imbalanced ones [41], allowing post-processing debiasing methods to improve fairness while better preserving predictive performance. Medical datasets typically exhibit extreme class imbalance along with varying degrees of unprivileged group representation, which can affect the effectiveness of debiasing methods. Table 5 presents the results of debiasing methods with the subset of class-unbalanced medical datasets, separately for those with either a lack (low representation) or an overrepresentation (high representation) of the unprivileged group. The results indicate that PSTA and ROC remain effective in these extreme cases, improving fairness while maintaining predictive performance. GSTAR, however, struggles, particularly when the unprivileged group is underrepresented. This can be attributed to its optimization process, which adjusts decision thresholds based on an estimated distribution that may be unreliable when limited data is available for this group. EPP and CPP still achieve competitive fairness improvements in these challenging conditions. This suggests that a hybrid approach could be beneficial, either through an ensemble method combining CPP and GSTAR, possibly alongside other methods, or by applying one method as a post-processing step after the other (e.g., using GSTAR after CPP). In contrast, FACT and MAB severely degrade predictive performance, suggesting that they might be unsuitable for medical applications with extreme class imbalance and unprivileged representation issues. Additionally, post-processing debiasing methods in medical datasets with roughly balanced class distributions and varying unprivileged representation levels exhibit (see Supplementary Table 5) effectiveness trends consistent with those observed in synthetic datasets at corresponding unprivileged representation levels in Fig. 5, where class distributions are perfectly balanced.

**Fig. 6:**
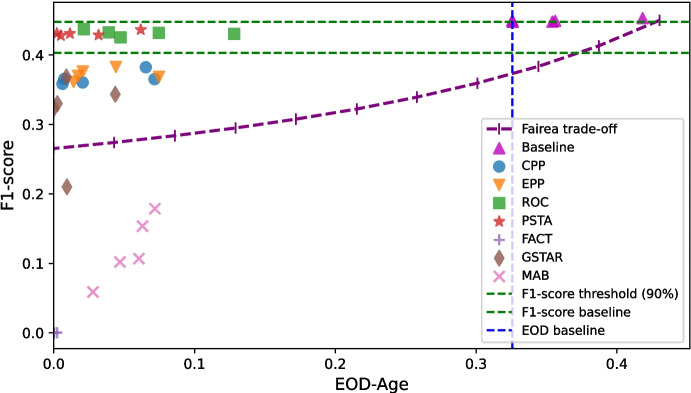
Fairea trade-off regions with the healthcare threshold for post-processing debiasing methods on a LR classifier applied to the Diabetes dataset, where Age is the protected attribute

**Fig. 7:**
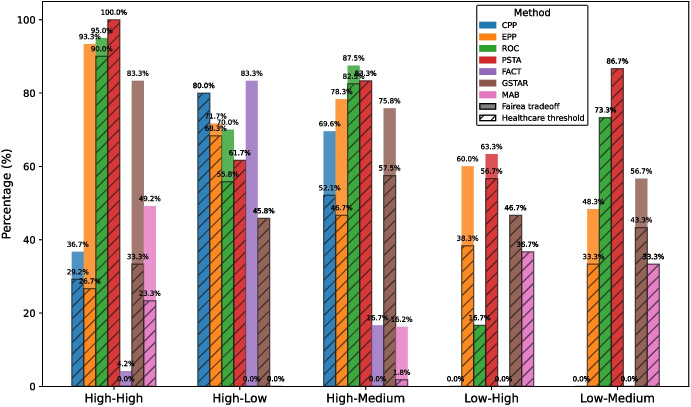
Proportion of cases surpassing the fairness-performance trade-off baseline (Fairea and healthcare threshold), stratified by class imbalance and unprivileged group representation across medical datasets. The *x*-axis categories follow the order (class imbalance - unprivileged representation); for example, “High-Low” indicates high class-imbalance and low representation of the unprivileged group

We have also compared the fairness and predictive performance of the debiasing methods in medical datasets with the baseline Fairea trade-off (Sect. 3.2.3). For example, Fig. 6 presents the results of the different methods (applied over an LR model) within the Fairea trade-off regions using the Diabetes dataset, Age as the protected attribute, F1-score as the performance metric, and EOD as the fairness metric. We consider every combination of *(medical dataset, protected attribute, ML model, fairness metric, and performance metric)* as a case and calculate the proportion of cases in which a debiasing method overcomes the baselines. A case is considered to overcome the baselines if it falls within the *win-win* or *good trade-off* regions. Figure 7 shows the proportion of cases in which each post-processing debiasing method overcomes the Fairea trade-off and the healthcare threshold, separated by class imbalance and unprivileged group representation. This separation allows us to examine how different levels of imbalance affect the effectiveness of debiasing methods in medical datasets. Among the post-processing methods studied, EPP and CPP stand out in cases of extreme class imbalance with a lack of unprivileged group representation, aligning with previous results for medical datasets.

Additionally, we examine the effectiveness of the debiasing methods across different ML models. These results, available as Supplementary Fig. 5, indicate that regardless of the ML modeling approach, the effectiveness trends of these methods remain consistent, aligning with findings from the synthetic data analyses.

### Type-2 Trade-off: Competing Fairness Metrics

We analyze the effect of debiasing methods on competing fairness metrics, focusing on the trade-off between EOp and EOd. Inspecting the results in Table 2, we can understand how post-processing debiasing methods influence these metrics. CPP targets EOp, aiming to equalize the true positive rates between protected and unprotected groups. However, it shows no change in EOp disparities and increases disparities in EOd. EPP focuses on EOd, aiming to balance false-positive and false-negative rates between protected and unprotected groups. It achieves notable reductions in disparities for both false-positive and false-negative rates across groups, thereby significantly improving EOd, while also enhancing EOp, ensuring a comprehensive approach to fairness. ROC targets EOp, effectively reducing disparities in both EOp and EOd, indicating a balanced approach to fairness. PSTA also focuses on EOp, achieving substantial reductions in EOp with moderate improvements in EOd. FACT aims at EOd but increases both EOd and EOp, leading to worsened unfair disparities. GSTAR targets EOd, effectively reducing both EOd and EOp. MAB aims to ensure accurate predictions across various subpopulations, mitigating systematic biases and going beyond traditional parity-based fairness notions such as EOp and EOd. It significantly reduces EOd and moderately reduces EOp.

Overall, methods like CPP and FACT may fail to reduce their target fairness metric and can worsen disparities in terms of other fairness metrics. Methods like ROC, PSTA, and GSTAR offer more balanced improvements across multiple metrics. MAB moderately improves fairness across subpopulations, contributing to more equitable outcomes.

### Bias Mitigation Influence on Untreated Attributes

**Fig. 8:**
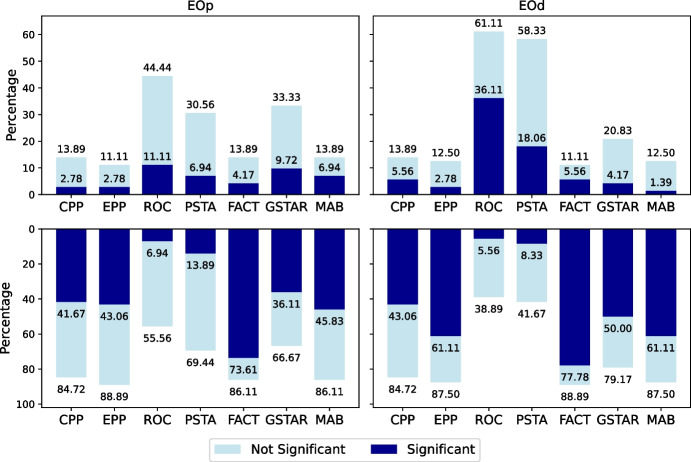
Summary of the impact of debiasing methods on untreated protected attributes for real-world datasets. The first row shows the proportion of cases where each debiasing method (applied to LR models), when targeting a given protected attribute, inadvertently increases the EOp and EOd measurements for other untreated protected attributes. The second row shows the proportion of cases where fairness metrics are inadvertently reduced. Dark-blue columns indicate the proportion of cases where the change is statistically significant, determined by considering a change with an absolute value of Cliff’s $\delta$ (effect size) greater than or equal to 0.428 as indicative of a large effect, whereas light-blue columns indicate non-significant changes

Debiasing methods, focused on mitigating bias for a specific protected attribute, might affect fairness concerning non-targeted protected attributes. We consider each combination of (dataset, treated protected attribute, non-targeted protected attribute) as a case and calculate the proportion of cases where a debiasing method, while addressing a specific protected attribute, leads to a fairness decrease or increase concerning other protected attributes (measured by changes in EOD or AOD metrics). Figure 8 shows the proportion of cases across datasets where each debiasing method results in fairness changes for non-targeted protected attributes. Regarding EOp, GSTAR and ROC show the highest percentages of unintended increases in unfair disparities (9.72% and 11.11% of cases, respectively). Regarding EOd, ROC and PSTA show the highest percentages (36.11% and 18.06% of cases, respectively). Although ROC and PSTA are among the most effective methods for improving fairness in treated attributes, they may unintentionally exacerbate disparities in untreated attributes. On the other hand, EPP, FACT, and MAB generally show substantial improvements regarding for both EOp and EOd concerning untreated attributes.

To better understand the mechanisms behind these unintended fairness reductions, it is important to examine how specific adjustments made by the debiasing methods contribute to disparities in other protected attributes. ROC, PSTA, and GSTAR are among the top three methods causing significant fairness reductions for unconsidered protected attributes. In ROC, predictions near the decision boundary are modified based on the targeted protected attribute (e.g., sex), relabeling low-confidence instances as positive for the unprivileged group (e.g., females) and negative for the privileged group (e.g., males). This adjustment affects outcomes for instances with intersecting privileged and unprivileged statuses, such as (Male, Non-White) and (Female, White), introducing further disparities for the untreated attribute (e.g., ethnicity). PSTA lowers the decision threshold for all members of the unprivileged group (e.g., females), resulting in positive predictions for individuals with scores between the new threshold (e.g., 0.4) and the original 0.5 threshold. If members of the subgroup (Female, White) receive more frequently prediction scores in this range compared to the subgroup (Male, Non-White), they will benefit more from this adjustment, contributing to race disparities. GSTAR applies separate thresholds for each targeted group (e.g., males and females), optimizing fairness for the considered attribute (sex) while minimizing classification error. However, the same threshold is applied within each group, without adjusting for other attributes (e.g., race). For instance, if GSTAR sets the threshold at 0.45 for females and 0.55 for males, members of the subgroup (Female, White) with prediction scores between 0.45 and 0.5 are classified as positive, while members of the subgroup (Male, Non-White) with scores between 0.5 and 0.55 are classified as negative, contributing to disparities in outcomes when race is unconsidered.

**Fig. 9:**
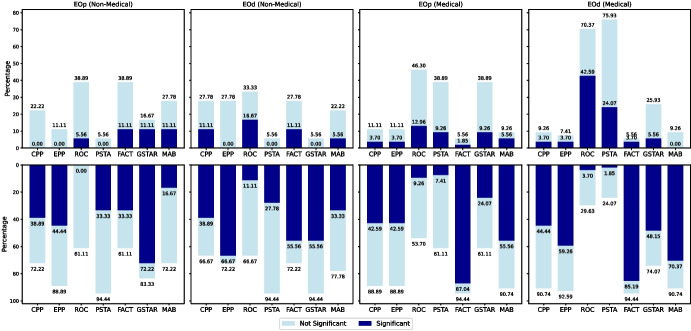
Summary of the impact of debiasing methods on untreated protected attributes for non-medical and medical datasets. The top row shows the proportion of undesired cases where each debiasing method (applied to LR models) inadvertently increases the values of EOp and EOd fairness metrics for untreated protected attributes, with results shown separately for non-medical and medical datasets. The bottom row shows the proportion of cases where fairness improves (EOp and EOd measurements are reduced). Dark-blue columns indicate the proportion of cases with statistically significant changes (Cliff’s $\delta \ge 0.428$), while light-blue columns represent non-significant changes, providing a detailed comparison of the effects across different dataset types

Figure 9 focuses on the impact of debiasing methods on untreated protected attributes separately for non-medical and medical datasets. Notably, the proportion of cases with a significant negative impact on untreated protected attributes is higher among medical datasets. To gain deeper insight into these fairness reductions, we investigate whether the adjustment strategies of debiasing methods and the underlying distribution patterns of protected attributes across datasets might play a role. We hypothesize that correlations between targeted and non-targeted protected attributes may influence how changes in fairness for a targeted protected attribute affect non-targeted protected attributes.

**Fig. 10:**
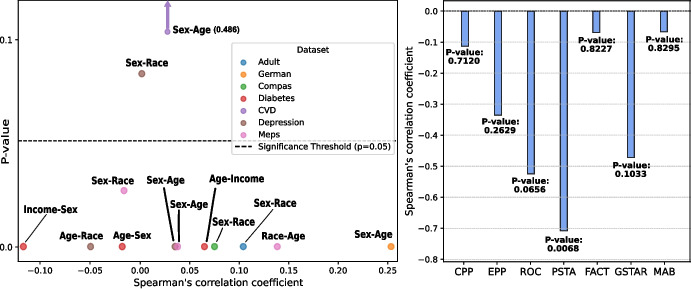
Pairwise Spearman correlation coefficients between protected attributes, with the horizontal dashed line representing the significance threshold (*p*-value = 0.05) (left). The Sex-Age (CVD) correlation value (0.486) falls outside the plot’s range and is marked with a purple arrow. Correlation between these coefficients and the proportion of fairness reductions for non-targeted protected attributes caused by debiasing methods (right)

Following the methodology in [14], we compute pairwise Spearman correlation coefficients between protected attributes across the eight datasets analyzed in this study. Figure 10 (first row) shows that, in health domain datasets, there is a higher prevalence of statistically significant negative correlations ($p-value < 0.05$) between targeted and non-targeted protected attributes compared to non-medical datasets. This indicates stronger inverse relationships between protected attributes in medical domains, which aligns with the results in Fig. 9. This observation supports our hypothesis that changes in fairness for one attribute may adversely affect another when they are inversely correlated. To test this hypothesis, we calculate the correlation between the Spearman coefficients of protected attributes and the proportion of cases where debiasing methods reduce fairness for non-targeted attributes. The results, presented in Fig. 10 (second row), align with our findings in this section, where ROC, PSTA, and GSTAR were identified as the top three methods causing significant fairness reductions for non-targeted protected attributes. PSTA stands out with a statistically significant and strong negative correlation (Corr: $-$0.7082, *p*-value: 0.0068), indicating that it may be the most effective method in reducing the percentage of worse cases caused by these inverse relationships. ROC and GSTAR show moderate negative correlations, with ROC approaching significance (Corr: $-$0.5247, *p*-value: 0.0656) and GSTAR displaying a moderate but not statistically significant correlation (Corr: $-$0.4721, *p*-value: 0.1033). These results suggest that the fairness reductions for untreated attributes observed with these methods may be partially explained by the inverse relationships between protected attributes within certain datasets, particularly in medical contexts. Other methods show no significance, aligning with their lower fairness reduction rates in EOp and EOd.

## Discussion

### Key Findings

#### Fairness-accuracy Trade-off

This study comprehensively evaluates seven post-proces-sing debiasing methods across nine real-world datasets as well as using synthetic data, allowing us to analyze their effectiveness in balancing fairness and accuracy across different types of ML models. PSTA and ROC emerge as the most balanced approaches, significantly reducing EOp and EOd disparities while maintaining BAcc and F1-score across different dataset conditions. GSTAR also performs well, particularly in datasets with a higher representation of the unprivileged group, providing substantial fairness improvements with moderate impacts on performance. Conversely, FACT and MAB severely degrade predictive performance, particularly in highly imbalanced settings. This would make them unsuitable for medical applications. CPP is effective in extreme class imbalance with low representation of the unprivileged group but struggles in balanced datasets. EPP achieves notable fairness improvements but at the cost of a significant drop in predictive performance. These findings reinforce the importance of selecting debiasing methods based on dataset characteristics to effectively manage fairness-performance trade-offs. Understanding these method-specific trade-offs is particularly crucial in high-stakes applications, such as healthcare, where choosing an appropriate debiasing method for the selected ML model can help mitigate disparities while preserving model reliability.

#### Influence of Classification Task’s Complexity

These findings are consistent with our analysis of the impact on the fairness of the classification task’s complexity, which used synthetic balanced datasets with controlled class separation. The results show that methods like PSTA and ROC perform effectively in tasks with lower class separation, where the classification task is more challenging. In these settings, they successfully balance fairness and accuracy. However, in less complex classification tasks with higher class separation, GSTAR achieves greater improvements in fairness without compromising performance. As the effectiveness of these methods might depend on the difficulty of the classification task, this should be taken into account when selecting debiasing methods. Task difficulty can be quantified using Bayes optimal classifiers, estimated via distance-based methods, nearest-neighbor methods, or classifier ensembles [4].

#### Influence on Untreated Attributes

Our analysis reveals that some debiasing methods, particularly GSTAR, ROC, and PSTA, can inadvertently and negatively influence fairness outcomes in untreated protected attributes. Although we have shown that these methods effectively improve fairness for the targeted attribute, they may lead to unintended unfair disparities in other untreated attributes. This unintended shift occurs because post-processing adjustments, such as decision boundary modifications and group-specific thresholding, do not account for interactions between multiple protected attributes. As a result, fairness disparities in unconsidered attributes become more pronounced. Furthermore, our findings indicate that negative correlations between protected attributes can amplify these unintended fairness shifts, reinforcing that a one-size-fits-all approach is insufficient. In cases of highly imbalanced datasets with low representation of the unprivileged group, CPP proves to be an effective alternative as it improves fairness while having less impact on untreated attributes. This makes CPP a viable option for medical settings, where extreme class imbalance is common, and fairness-sensitive decisions are crucial. These findings underscore the need for careful method selection based on specific application contexts.

### Limitations

While our empirical study provides a systematic evaluation of post-processing debiasing methods in healthcare, certain limitations should be acknowledged. The selection of debiasing methods was based on availability and prior effectiveness reported in the literature. However, newer or less commonly studied methods could yield different results. Furthermore, our study highlights that debiasing one protected attribute can introduce disparities in others, yet fairness-aware multi-attribute interventions were not explored. Addressing intersectional biases effectively remains an open challenge that requires further research. Another important consideration is that the datasets used cannot capture all the possible real-world populations, contexts, and biases in healthcare. Although we included medical real-world datasets, along with real-world datasets from other domains and synthetic data to ensure systematic testing, the study may still lack a broader representation of scenarios. We have replicated some of these with synthetic data but, while useful for controlled experiments, it does not reproduce the complexity of real-world medical data. Finally, our evaluation was limited to structured tabular healthcare data, excluding other important modalities such as medical imaging, electronic health records, and genomic data.

## Conclusion and Future Recommendations

This study highlights the importance of selecting appropriate post-processing debiasing methods based on data characteristics. Methods like PSTA and ROC consistently improve fairness while maintaining predictive performance, while others like GSTAR or CPP are more sensitive to unbalanced data distribution. Our findings emphasize that negative correlations between protected attributes can amplify unintended fairness disparities. Furthermore, post-processing debiasing methods are model-agnostic, allowing them to be applied across various classification models without modifying the learning process. Our results indicate that data characteristics have a greater impact on fairness outcomes than the choice of classifier. Regardless of whether the model is a linear model, a tree-based ensemble, or a deep neural network, the effectiveness of debiasing methods follows consistent trends, reinforcing the importance of dataset properties when selecting fairness interventions.

Future research should explore adaptive fairness strategies that dynamically adjust debiasing methods based on dataset properties, ensuring stability across different representation levels. Additionally, combining debiasing methods in hybrid approaches could be a promising direction, leveraging their complementary strengths to mitigate fairness-performance trade-offs. While the present study does not directly implement such hybrid methods, it provides insights that could inform future research on integrating multiple debiasing techniques for improved fairness in clinical settings.

Investigating multi-attribute fairness optimization can help mitigate trade-offs between treated and untreated attributes. Additionally, integrating causal fairness analysis may provide deeper insights into how fairness interventions propagate across correlated attributes, leading to more robust and equitable ML models in high-stakes domains like healthcare.

## Supplementary Information

Below is the link to the electronic supplementary material.

Supplementary file 1 (pdf 381 KB)

## Data Availability

All the datasets are publicly available. Detailed accessibility conditions for real-world datasets, as well as source code for synthetic data generation, are available at https://github.com/ngoc-vien-dang/FairML4H-PostProcessing.
