# Supplementary material for: Empirical Comparison of Post-processing Debiasing Methods for Machine Learning Classifiers in Healthcare
Source: J Healthc Inform Res. 2025 Mar 20;9(3):465–93. doi: 10.1007/s41666-025-00196-7 (PMC12290158; doi:10.1007/s41666-025-00196-7)
Supplement: Supplementary file 1 — (pdf 381 KB) [file 41666_2025_196_MOESM1_ESM.pdf]

Supplementary Material

(a) Low Separation

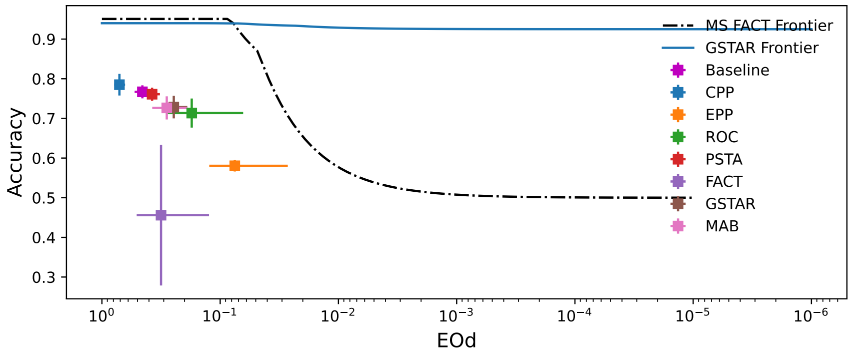

(b) Medium Separation

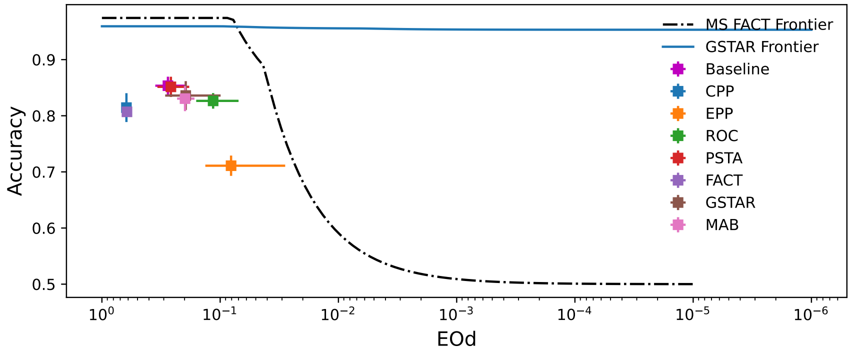

(c) High Separation

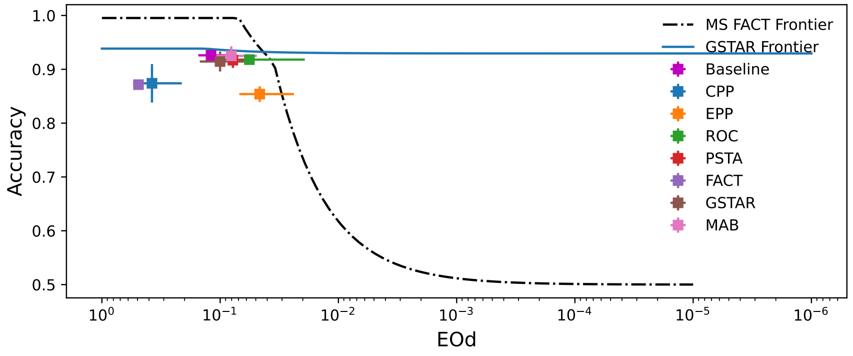

Supplementary Figure 1: Pareto frontiers of accuracy and fairness trade-offs for post-processing debiasing methods under different class separation levels on synthetic data, using XGB as the classifier. Points represent mean Acc-EOd and error bars indicate the standard deviation over 5-fold cross-validation.

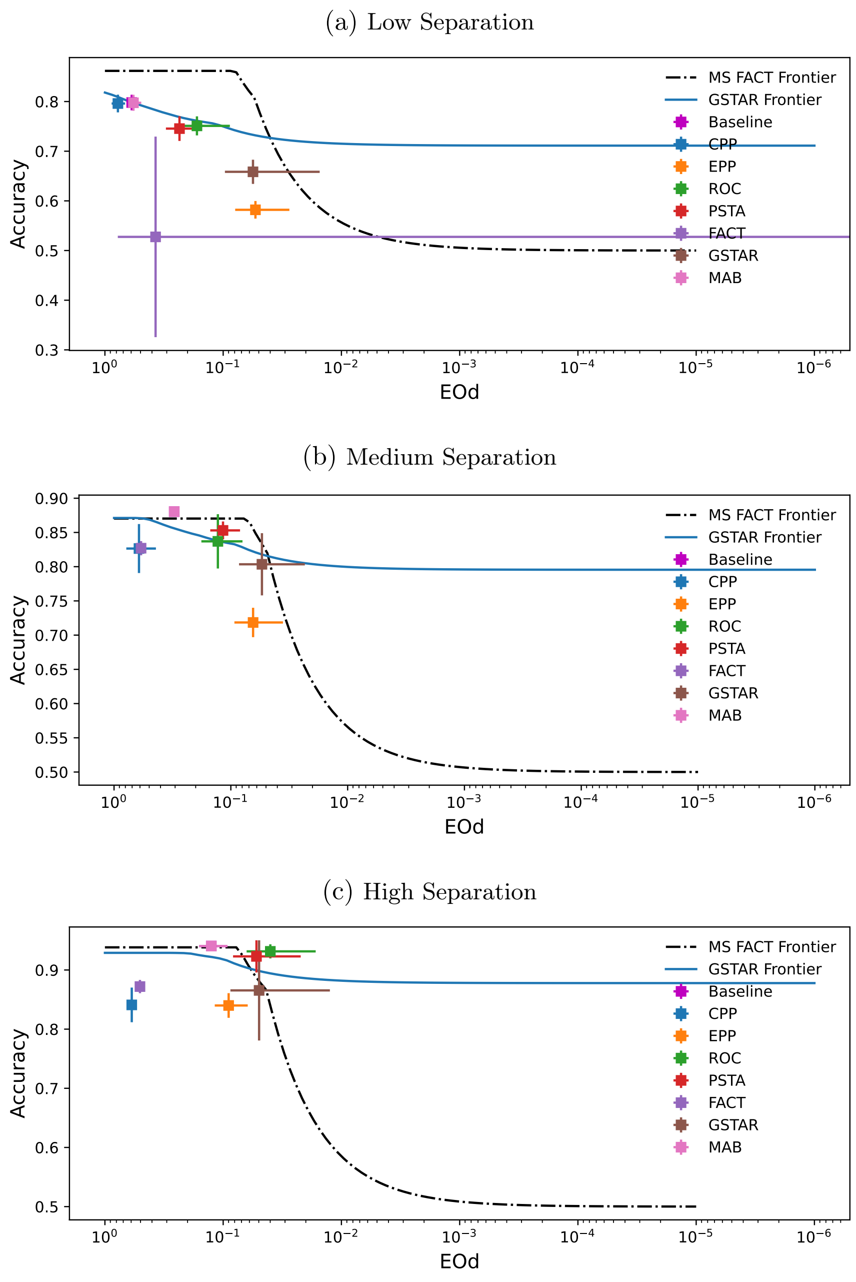

Supplementary Figure 2: Pareto frontiers of accuracy and fairness trade-offs for post-processing debiasing methods under different class separation levels on synthetic data, using DNN as the classifier. Points represent mean Acc-EOd and error bars indicate the standard deviation over 5-fold cross-validation.

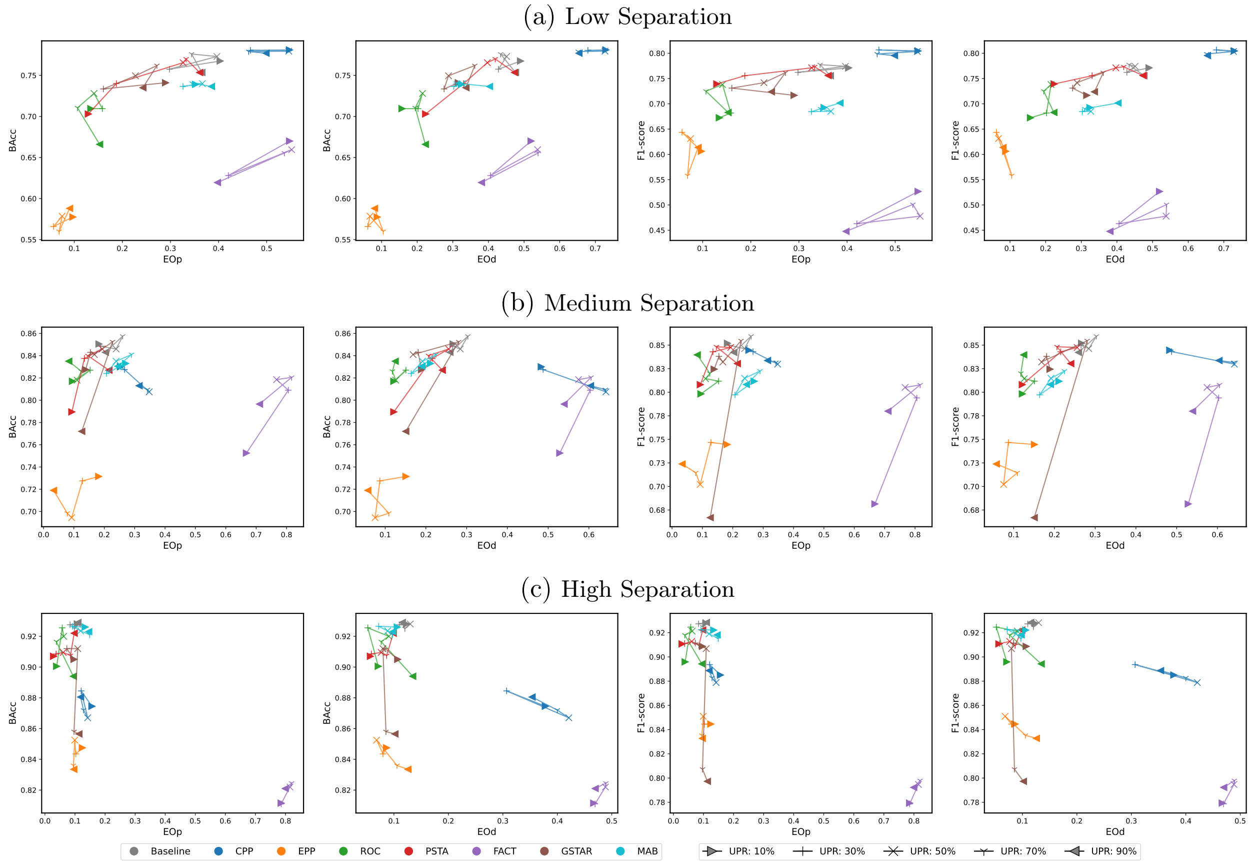

Supplementary Figure 3: Type-1 trade-offs between performance metrics (BAcc and F1-score) and fairness metrics (EOp and EOd) achieved by post-processing debiasing methods across synthetic datasets with varying levels of unprivileged representation (10%, 30%, 50%, 70%, 90%; lines connect the results of similar experiments with increasingly higher UGR) and class separation (low, medium, high; plots in different rows), using XGB as the classifier.

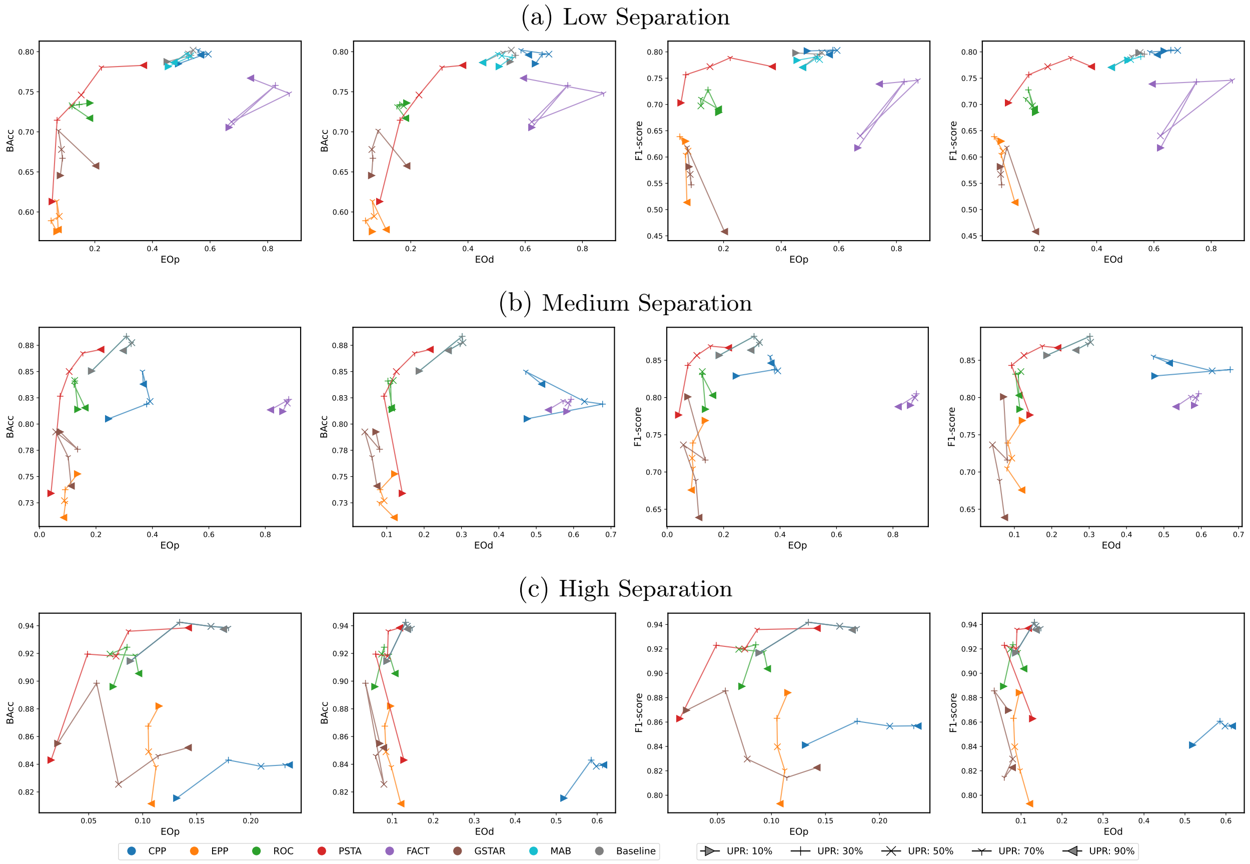

Supplementary Figure 4: Type-1 trade-offs between performance metrics (BAcc and F1-score) and fairness metrics (EOp and EOd) achieved by post-processing debiasing methods across synthetic datasets with varying levels of unprivileged representation (10%, 30%, 50%, 70%, 90%; lines connect the results of similar experiments with increasingly higher UGR) and class separation (low, medium, high; plots in different rows), using DNN as the classifier.

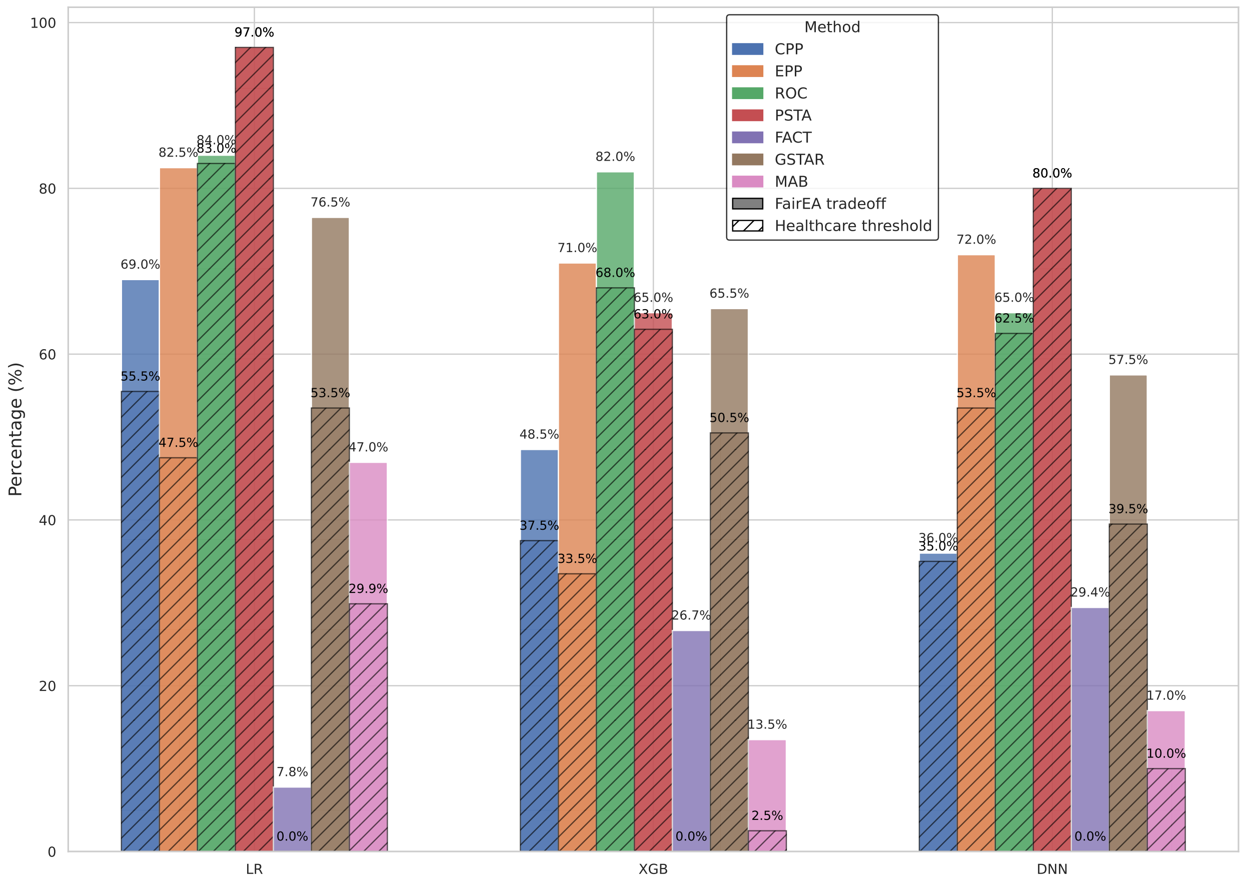

Supplementary Figure 5: Proportion of cases surpassing the fairness-performance trade-off baseline (Fairea and healthcare threshold), stratified by ML models across medical datasets.

Supplementary Table 1: Protected attributes and other relevant features of the used non-medical datasets.

**Subgroup**	**Attribute**	**Adult**	**German Credit**	**COMPAS**	**Bank**
	No. of subjects	48,842	1,000	6,172	41,188
	No. of features	13	20	8	21
**Sex (%);FCP (%)**	Male	66.8 (30.4)	69.0 (72.3)	81.0 (52.1)	-
	Female	33.2 (10.9)	31.0 (64.8)	19.0 (64.9)	-
**Age (%);FCP (%)**	<25 years	-	19.0 (57.9)	-	-
	25+ years	-	81.0 (72.8)	-	-
	<25, 60+ years	-	-	-	5.5 (32.2)
	25-60 years	-	-	-	94.5 (10.0)
**Race (%);FCP (%)**	White	85.5 (25.4)	-	34.1 (60.9)	-
	Other	14.5 (15.3)	-	65.9 (51.2)	-

Supplementary Table 2: Protected attributes and other relevant features of the used medical datasets.

**Subgroup**	**Attribute**	**Diabetes**	**CVD**	**MEPS**	**Depression**
	No. of subjects	229,474	920	15,785	461,033
	No. of features	22	15	140	144
**Sex (%);FCP (%)**	Male	-	78.9 (63.2)	47.9 (13.2)	46.6 (3.2)
	Female	-	21.1 (25.8)	52.1 (20.7)	53.4 (4.5)
**Age (%);FCP (%)**	18-44 years	21.3 (4.5)	-	-	-
	45+ years	78.7 (18.2)	-	-	-
	<65 years	-	88.8 (53.5)	0.88 (12.9)	80.5 (3.9)
	65+ years	-	11.2 (69.9)	0.12 (49.5)	19.5 (4.0)
**Race (%);FCP (%)**	White	-	-	64.3 (12.5)	6.1 (3.7)
	Other	-	-	35.7 (25.5)	93.9 (3.9)
**Income (%);FCP (%)**	Low-income	51.3 (19.4)	-	-	-
	High-income	48.7 (11.0)	-	-	-

Supplementary Table 3: Implementation details of ML models and hyperparameter selection of the used non-medical datasets.

Dataset	Model	Hyper-parameter
Adult	LR	L2-regularized, solver: ['lbfgs'], c_values: [1.0], class_weight: ['balanced']
	XGB	'max_depth': [6], 'learning_rate': [0.3], 'min_child_weight': [1], 'subsample': [1], 'scale_pos_weight': [neg/pos sample ratio in y_train]
	DNN	'hidden_layer_sizes': (64, 32, 16, 8, 4), 'learning_rate_init': 0.001, 'solver': 'adam', 'early_stopping': True, 'alpha': 1e-3, 'max_iter': 200
German Credit	LR	L2-regularized, solver: ['lbfgs'], c_values: [1.0], class_weight: ['balanced']
	XGB	'max_depth': [6], 'learning_rate': [0.3], 'min_child_weight': [1], 'subsample': [1], 'scale_pos_weight': [neg/pos sample ratio in y_train]
	DNN	'hidden_layer_sizes': (64, 32, 16, 8, 4), 'learning_rate_init': 0.001, 'solver': 'adam', 'early_stopping': True, 'alpha': 1e-3, 'max_iter': 200
COMPAS	LR	L2-regularized, solver: ['lbfgs'], c_values: [1.0]
	XGB	'max_depth': [6], 'learning_rate': [0.3], 'min_child_weight': [1], 'subsample': [1]
	DNN	'hidden_layer_sizes': (64, 32, 16, 8, 4), 'learning_rate_init': 0.001, 'solver': 'adam', 'early_stopping': True, 'alpha': 1e-3, 'max_iter': 200
Bank Marketing	LR	L2-regularized, solver: ['lbfgs'], c_values: [1.0]
	XGB	'max_depth': [6], 'learning_rate': [0.3], 'min_child_weight': [1], 'subsample': [1]
	DNN	'hidden_layer_sizes': (64, 32, 16, 8, 4), 'learning_rate_init': 0.001, 'solver': 'adam', 'early_stopping': True, 'alpha': 1e-3, 'max_iter': 200

Supplementary Table 4: Implementation details of ML models and hyperparameter selection of the used medical datasets.

Dataset	Model	Hyper-parameter
Diabetes	LR	L2-regularized, solver: ['lbfgs'], c_values: [1.0], class_weight: ['balanced']
	XGB	'max_depth': [6], 'learning_rate': [0.3], 'min_child_weight': [1], 'subsample': [1], 'scale_pos_weight': [neg/pos sample ratio in y_train]
	DNN	'hidden_layer_sizes': (80,), 'learning_rate_init': 0.001, 'solver': 'adam', 'early_stopping': True, 'alpha': 1e-3, 'max_iter': 200
CVD	LR	L2-regularized, solver: ['lbfgs'], c_values: [1.0]
	XGB	'max_depth': [6], 'learning_rate': [0.3], 'min_child_weight': [1], 'subsample': [1]
	DNN	'hidden_layer_sizes': (128, 64, 32, 16), 'learning_rate_init': 0.001, 'solver': 'adam', 'early_stopping': True, 'alpha': 1e-3, 'max_iter': 200
MEPS	LR	L2-regularized, solver: ['lbfgs'], c_values: [1.0], class_weight: ['balanced']
	XGB	'max_depth': [6], 'learning_rate': [0.3], 'min_child_weight': [1], 'subsample': [1], 'scale_pos_weight': [neg/pos sample ratio in y_train]
	DNN	'hidden_layer_sizes': (64, 32, 16, 8, 4), 'learning_rate_init': 0.001, 'solver': 'adam', 'early_stopping': True, 'alpha': 1e-3, 'max_iter': 200
Depression	LR	L2-regularized, solver: ['lbfgs'], c_values: [1.0], class_weight: ['balanced']
	XGB	'max_depth': [6], 'learning_rate': [0.3], 'min_child_weight': [1], 'subsample': [1], 'scale_pos_weight': [neg/pos sample ratio in y_train]
	DNN	'hidden_layer_sizes': (64, 32, 16, 8, 4), 'learning_rate_init': 0.001, 'solver': 'sgd', 'early_stopping': False, 'alpha': None, 'max_iter': 10, 'class_weight': 'balanced'

Supplementary Table 5: Mean absolute and relative (in parentheses) changes –before and after debiasing using the different debiasing methods– in performance and fairness metrics averaged across ML models for medical datasets with roughly balanced class distributions, separated by unprivileged representation degree. The top three values for each metric are highlighted.

	**Unprivileged representation degree (High)**				**Unprivileged representation degree (Medium)**			
**Method**	**F1-score**	**BAcc**	**EOp**	**EOd**	**F1-score**	**BAcc**	**EOp**	**EOd**
CPP	-0.007 (-0.9%)	-0.013 (-1.6%)	0.026 (25.3%)	0.241 (164.5%)	-0.047 (-5.7%)	-0.123 (-15.3%)	0.105 (50.6%)	0.315 (149.8%)
EPP	-0.050 (-6.1%)	-0.098 (-12.2%)	-0.032 (-29.8%)	-0.057 (-32.5%)	-0.113 (-13.7%)	-0.083 (-10.3%)	-0.072 (-36.0%)	-0.110 (-52.3%)
ROC	-0.011 (-1.4%)	-0.007 (-0.8%)	0.020 (21.0%)	-0.006 (-1.1%)	-0.011 (-1.3%)	-0.015 (-1.8%)	-0.097 (-47.1%)	-0.087 (-40.9%)
PSTA	-0.010 (-1.3%)	-0.035 (-4.4%)	-0.011 (-9.6%)	-0.041 (-24.6%)	-0.009 (-1.0%)	-0.022 (-2.7%)	-0.129 (-63.3%)	-0.115 (-54.5%)
FACT	-0.091 (-11.0%)	-0.058 (-7.2%)	0.511 (482.5%)	0.256 (164.7%)	-0.068 (-8.2%)	-0.061 (-7.6%)	0.303 (147.4%)	0.094 (44.7%)
GSTAR	-0.065 (-7.9%)	-0.032 (-4.0%)	-0.021 (-19.8%)	-0.018 (-10.5%)	-0.117 (-14.1%)	-0.054 (-6.6%)	-0.088 (-42.3%)	-0.107 (-50.2%)
MAB	-0.028 (-3.4%)	-0.008 (-1.1%)	0.023 (23.2%)	-0.003 (-1.7%)	-0.028 (-3.4%)	-0.008 (-1.1%)	-0.012 (-5.7%)	-0.013 (-6.6%)
Average	-0.037 (-4.5%)	-0.036 (-4.5%)	0.074 (70.4%)	0.053 (37.0%)	-0.056 (-6.8%)	-0.052 (-6.5%)	0.001 (0.5%)	-0.003 (-1.4%)
